# Supplementary material for: Enhancer adoption by an LTR retrotransposon generates viral-like particles, causing developmental limb phenotypes
Source: Nat Genet. 2025 Jul 9;57(7):1766–76. doi: 10.1038/s41588-025-02248-5 (PMC12283350; doi:10.1038/s41588-025-02248-5)
Supplement: Supplementary file 1 — Supplementary notes and references, Supplementary Table legends, Supplementary methods and references. [file 41588_2025_2248_MOESM1_ESM.pdf]

nature genetics

Supplementary information

https://doi.org/10.1038/s41588-025-02248-5

Enhancer adoption by an LTR retrotransposon generates viral-like particles, causing developmental limb phenotypes

In the format provided by the authors and unedited

Supplementary information

SUPPLEMENTARY NOTES 2

SUPPLEMENTARY NOTES REFERENCES 2

SUPPLEMENTARY TABLE LEGENDS 3

Supplementary Table 1 | List of single-cell RNA-seq marker genes. 3

Supplementary Table 2 | Rpkm gene expression from bulk RNA-sequencing from early E11.5 forelimbs.. 3

Supplementary Table 3 | List of primers used for this study. 3

Supplementary Table 4 | List of sgRNAs used for this study. 3

Supplementary Table 5 | List of homology arms mm10 coordinates used for this study. 3

Supplementary Table 6 | List of off-targets for the sgRNA Deletion Dac1J-Pol-5'. 3

Supplementary Table 7 | List of WISH probes used for this study 3

SUPPLEMENTARY METHODS 4

RNA-sequencing data processing 4

single-cell RNA-sequencing data processing 4

Capture Hi-C data processing 4

4C-seq data processing 5

ChIP-sequencing data processing 5

CTCF binding site motif detection 5

Micro-computed tomography 5

Whole-mount in situ hybridization (WISH) 5

Hybridization-chain-reaction (HCR) RNA fluorescent *in situ* 5

Lysotracker staining 6

SUPPLEMENTARY METHODS REFERENCE 6

Supplementary Notes

1. We identified a loss of 82.6% of ectoderm cells in the *Dac1J*/*Dac1J* E11.5 limbs compared to wild-type: 3.7% and 0.7% dorso-ventral ectoderm and 0.9% and 0.1% AER cells in wild-type and *Dac1J*/*Dac1J*, respectively (**Fig.2c**).

2. The activation of the *Fbxw4* gene, located ~ 10kb upstream of the MusD-*Dac1J* insertion and previously proposed to be involved in Dactylaplasia[1], was not affected (**Extended data Fig. 3c and 4c**). We have previously shown that ectopic expression of *Lbx1* and *Btrc* is involved in the patho-mechanism of the human split-hand-food malformation type 3 (SHFM3)[2]. In *Dac1J*/*Dac1J* embryos, these two genes were also properly expressed in all cell types from E9.5 to E11.5 (**Extended data Fig. 4c**).

3. Of note, an architectural stripe[3] was observed in the *Dac1J* mutant (**Fig. 3d**) but not in the 5LTR-LacZ-KI (**Fig. 3h**).

4. At least nine complete MusD copies with full coding potential exist in the mouse genome, including three shown to be autonomous proviruses still functional for retro-transposition[4]. Sequence alignment revealed that the *Dac1J* GAG and POL protein sequences were almost identical to the three autonomous MusD (**Extended data Fig. 6a**)

5. Of note, no limb staining was observed in the 5'LTR-LacZ-KI at the *Shh* locus, which we attributed to the position of the knock-in as suggested by data from *Symmons et al.*[5].

Supplementary Notes References

1. Sidow, A. *et al.* A novel member of the F-box/WD40 gene family, encoding dactylin, is disrupted in the mouse dactylaplasia mutant. *Nat. Genet.* **23**, 104–107 (1999).
2. Cova, G. *et al.* Combinatorial effects on gene expression at the Lbx1/Fgf8 locus resolve split-hand/foot malformation type 3. *Nat. Commun.* **14**, 1–17 (2023).
3. Kraft, K. *et al.* Serial genomic inversions induce tissue-specific architectural stripes, gene misexpression and congenital malformations. *Nat. Cell Biol.* **21**, 305–310 (2019).
4. Ribet, D., Dewannieux, M. & Heidmann, T. An active murine transposon family pair: Retrotransposition of 'master' MusD copies and ETn trans-mobilization. *Genome Res.* **14**, 2261–2267 (2004).
5. Symmons, O. *et al.* The Shh Topological Domain Facilitates the Action of Remote Enhancers by Reducing the Effects of Genomic Distances. *Dev. Cell* **39**, 529–543 (2016).

Supplementary Table legends

Supplementary Table 1 | List of single-cell RNA-seq marker genes.
p-value was calculated using a Wilcoxon Rank Sum test (Seurat package). Adj *p*-values are based on Bonferroni correction. Genes from in Extended data Figure 3a are shown in bold.

Supplementary Table 2 | Rpkm gene expression from bulk RNA-sequencing from early E11.5 forelimbs.

Supplementary Table 3 | List of primers used for this study.

Supplementary Table 4 | List of sgRNAs used for this study.

Supplementary Table 5 | List of homology arms mm10 coordinates used for this study.

Supplementary Table 6 | List of off-targets for the sgRNA Deletion Dac1J-Pol-5'.

Supplementary Table 7 | List of WISH probes used for this study.

Supplementary methods

RNA-sequencing data processing

Reads were mapped to the mouse reference genome (*mm10*) using the STAR mapper[1] (splice junctions based on RefSeq; options: –alignIntronMin 20–alignIntronMax 500000–outFilterMultimapNmax 5–outFilterMismatchNmax 10–outFilterMismatchNoverLmax 0.1). Reads were subsequently used for expression analysis via the Cufflinks package[2] (version 2.2.1; default settings). Transcripts of each sample were assembled using Cufflinks provided with reference gene annotations from Ensembl. The resulting assemblies were then merged via Cuffmerge. The cut-off for significantly altered gene expression was an adjusted *p*-value of 0.05. Differential gene expression was ascertained using the DESeq2 package. Heatmap results were visualized with the R package pheatmap.

single-cell RNA-sequencing data processing

Computational analysis of the sequenced samples was done with Cell Ranger and the Seurat package v.3 (10x Genomics lnc.). Reads were mapped to the mm10 transcriptome customized with an extra chromosome containing the *Dac1J* sequence. Mapping and preprocessing were done with Cell Ranger default parameters version 3.0.2. Genes expressed in fewer than 10 cells were filtered out. Cells were filtered depending on the level of percentage of UMIs mapping to mitochondrial genes, the number of expressed genes, and library size. Only cells with more than 500 detected genes and less than 15% of mitochondrial UMI counts were considered for downstream analysis. When checking for the presence of confounding factors we identified both cell cycle and sex confounding effects. The presence of a cell-cycle effect was checked by using a principal component analysis (PCA) on a set of G2/M and S phase markers genes. We estimated the phase of each cell by assigning a score based on the cell expression of G2/M and S phase markers using the "CellCycleScoring" Seurat function. We used the difference between G2M and S phase scores as a confounding effect in order to only correct for cell cycle phase among proliferating cells while maintaining the difference between stem and progenitor cells as recommended when studying differentiating processes. UMI counts were normalized using scTransform[3], regressing out the difference between G2M and S phase score and the percentage of UMIs mapping to *Xist* to correct for the difference of ratio between female and male embryos among experiments. We built a common latent cell representation across samples by integrating the sample-wise top 50 cell principal components based on the tap 1000 highly variable genes using the Seurat CCA method[4]. The top 50 principal components of this joint integrated assay were used for the visualization of the Uniform Manifold Approximation and Projection (UMAP). We clustered cells by first constructing a Shared Nearest Neighbor (SNN) Graph based on the Euclidean distance in the first 20 integrated principal components space using the "FindNeighbors" function with k.param set to 20. Cell clusters were defined using the Louvain algorithm as a modularity optimization technique implemented in the function "FindCluster" with the resolution parameter set to 0.2. Visualization of gene expression was computed after a new scTransform normalization run on the merged raw count assays regressing out for cell cycle and sex effect as previously described. For each cluster, conserved markers between mutant and wild types were identified using the Seurat "FindConservedMarkers" function and were then used for cell-type annotation.

Capture Hi-C data processing

Paired-end fastq reads were first trimmed to 50bp to have the same read length across all sequencing runs before alignment to mm10 as well as the appropriate custom mm10 genomes incorporating either the *Dac1J* or the 5LTR-LacZ insertions. Read mapping and further filtering for each library were carried out separately using the HiCUP pipeline v0.8.3[5] with Bowtie2 v2.5.0[6] as the aligner and no size selection or filling. Binned and Knight-Ruiz (KR) normalized contact matrices from merged biological replicates

were then generated with Juicer tools v1.22.0[7] for the region of interest (*chr19: 45,100,000-45,900,000*, MAPQ ≥ 30). Before computing subtraction maps, Hi-C matrices from wild-type samples mapped to the appropriate custom genomes were scaled to half that of corresponding heterozygous mutants (*Dac1J*-Bl6 & 5LTR-LacZ) to account for the wild-type allele. Subtraction between KR-normalized matrices (i.e. mutant - wild-type) was then calculated as previously described[8,9]. Specifically, the coverage of the two maps was equalized before element-wise subtraction and z-scaled within the same subdiagonal. Subtraction and matrices visualization at 5kb resolution were done using a custom Python script via the FANC Python API[10].

4C-seq data processing
4C-seq processing was performed as previously described[11]. Reads were pre-processed and mapped to the mm10 reference genome using BWA. The viewpoint and adjacent fragments 1.5 kb upstream and downstream were removed and a window of 10 fragments was chosen to normalize the data per million mapped reads (RPM). To visualize the data, we created files in bedGraph track format for the read counts of each fragment or in a specified window of fragments.

ChIP-sequencing data processing
Reads were mapped to the mouse reference genome (*mm10*) using bowtie2 mapper. SAMtools[12] was used for filtering, sorting, and removing duplicates, and deepTools[13] for generating coverage tracks.

CTCF binding site motif detection
The FIMO (Find Individual Motif Occurrences), MEME suite 5.5.4 (https://meme-suite.org/meme/tools/fimo) was used to detect CTCF motif in the *Dac1J* sequence (AB305072).

Micro-computed tomography
Autopods of 7-months-old wild type and *Dac1J*/*Dac1J* (*n*= 2 per genotype) were wrapped in plastic film and scanned *ex vivo* using a SkyScan 1172 high-resolution micro-computed tomography system (Brucker microCT) at 10-µm resolution, 80 kV, and 124µA. 3D model reconstruction was done with the SkyScan image analysis software (computed tomography analyser and computed tomography volume) (Brucker microCT).

Whole-mount in situ hybridization (WISH)
RNA expression in mouse embryos from wild-type and mutants was assessed by WISH using digoxigenin (DIG)-labeled antisense riboprobes for *Fgf8, Lbx1, Shh, and Sox9* transcribed from linearized gene-specific probes (PCR DIG Probe Synthesis Kit; Roche, Cat. #11636090910). Primers for probe generation are listed in **Supplementary Table 7**. Embryos were collected and fixed overnight in 4% PFA in PBS, then washed twice for 30min in PBS with 0.1% Tween (PBST), dehydrated for 30 min each in 25%, 50%, and 75% methanol in PBST, and stored at −20 °C in 100% methanol. WISH was performed as previously described[14].

Hybridization-chain-reaction (HCR) RNA fluorescent *in situ*
HCR RNA-FISH was performed as previously described[15] using the kit from *Molecular Instruments*. Briefly, mouse embryos were dissected in 1x PBS and fixed overnight in 4% PFA in PBS, then washed twice for 30min in PBS with 0.1% Tween (PBST), dehydrated for 30 min each in 25%, 50%, and 75% methanol in PBST, and stored at −20 °C in 100% methanol until use. When starting the HCR protocol, embryos were first rehydrated in methanol/PBST (75%, 50%, 25%), then washed in PBST, treated with 10ug/mL proteinase K solution for 15 minutes, washed in PBST, postfixed in 4% PFA in PBS and washed again in PBST. For the detection stage, 1-4 embryos were transferred in 2mL tubes, incubated

with pre-hybridization buffer (room temperature), and in probe hybridization buffer (37°C). Hybridization with the *Fgf8* probe (ordered from *Molecular Instruments*) was done overnight at 37°C. The day after, embryos were washed with the wash buffer at 37°C and with 5x SSCT at room temperature. To visualize the probes, embryos were incubated with the amplification buffer and 30pmol of each hairpin (h1 and h2) overnight at room temperature. On the last day, embryos were washed several times with 5x SSCT at room temperature. Prior to clearing, embryos were washed three times with 0.02 M phosphate buffer (PB, 0.025M NaH_2PO_4, and 0.075M Na_2HPO_4, pH 7.4). The clearing was performed by incubation in RIMS (13% Histodenz (Sigma-Aldrich D2158) in 0.02M PB) at 4 °C for at least one day. Forelimbs were then imaged with a Zeiss LSM880 confocal laser-scanning microscope in LSM mode.

Lysotracker staining

E12.5 embryos were collected in ice-cold PBS and forelimbs were microdissected. Forelimbs were washed once with PBS and then incubated with a 2μM Lysotracker solution (xx) for 30 minutes at 37°C. After incubation, limbs were washed 3 times with PBS and then fixed overnight in PFA 4%. The limbs were then cleared with RIMS and imaged with a Zeiss LSM880 confocal laser-scanning microscope.

Supplementary methods Reference

1. Dobin, A. *et al.* STAR: ultrafast universal RNA-seq aligner. *Bioinformatics* **29**, 15–21 (2013).
2. Trapnell, C. *et al.* Differential gene and transcript expression analysis of RNA-seq experiments with TopHat and Cufflinks. *Nat. Protoc.* **7**, 562–578 (2012).
3. Hafemeister, C. & Satija, R. Normalization and variance stabilization of single-cell RNA-seq data using regularized negative binomial regression. *Genome Biol.* **20**, 296 (2019).
4. Stuart, T. *et al.* Comprehensive Integration of Single-Cell Data. *Cell* **177**, 1888-1902.e21 (2019).
5. Wingett, S. *et al.* HiCUP: pipeline for mapping and processing Hi-C data. *F1000Research* **4**, 1310 (2015).
6. Langmead, B. & Salzberg, S. L. Fast gapped-read alignment with Bowtie 2. *Nat. Methods* **9**, 357–359 (2012).
7. Durand, N. C. *et al.* Juicer Provides a One-Click System for Analyzing Loop-Resolution Hi-C Experiments. *Cell Syst.* **3**, 95–98 (2016).
8. Bianco, S. *et al.* Polymer physics predicts the effects of structural variants on chromatin architecture. *Nat. Genet.* **50**, 662–667 (2018).
9. Anania, C. *et al.* In vivo dissection of a clustered-CTCF domain boundary reveals developmental principles of regulatory insulation. *Nat. Genet.* **54**, 1026–1036 (2022).
10. Kruse, K., Hug, C. B. & Vaquerizas, J. M. FAN-C: a feature-rich framework for the analysis and visualisation of chromosome conformation capture data. *Genome Biol.* **21**, 303 (2020).
11. Lupiáñez, D. G. *et al.* Disruptions of topological chromatin domains cause pathogenic rewiring of gene-enhancer interactions. *Cell* **161**, 1012–1025 (2015).
12. Li, H. *et al.* The Sequence Alignment/Map format and SAMtools. *Bioinformatics* **25**, 2078–2079 (2009).
13. Ramírez, F. *et al.* deepTools2: a next generation web server for deep-sequencing data analysis. *Nucleic Acids Res.* **44**, W160-5 (2016).
14. Cova, G. *et al.* Combinatorial effects on gene expression at the Lbx1/Fgf8 locus resolve split-hand/foot malformation type 3. *Nat. Commun.* **14**, 1–17 (2023).
15. Choi, H. M. T. *et al.* Third-generation in situ hybridization chain reaction: multiplexed, quantitative, sensitive, versatile, robust. *Development* **145**, dev165753 (2018).
