# Supplementary material for: Enhancer adoption by an LTR retrotransposon generates viral-like particles, causing developmental limb phenotypes
Source: Nat Genet. 2025 Jul 9;57(7):1766–76. doi: 10.1038/s41588-025-02248-5 (PMC12283350; doi:10.1038/s41588-025-02248-5)
Supplement: Supplementary file 2 — Reporting Summary [file 41588_2025_2248_MOESM2_ESM.pdf]

nature portfolio

Corresponding author(s): Stefan Mundlos, Juliane Glaser

Last updated by author(s): 13/03/2025

Reporting Summary

Nature Portfolio wishes to improve the reproducibility of the work that we publish. This form provides structure for consistency and transparency in reporting. For further information on Nature Portfolio policies, see our Editorial Policies and the Editorial Policy Checklist.

Statistics

For all statistical analyses, confirm that the following items are present in the figure legend, table legend, main text, or Methods section.

n/a	Confirmed	
☐	☒	The exact sample size (*n*) for each experimental group/condition, given as a discrete number and unit of measurement
☐	☒	A statement on whether measurements were taken from distinct samples or whether the same sample was measured repeatedly
☐	☒	The statistical test(s) used AND whether they are one- or two-sided<br>*Only common tests should be described solely by name; describe more complex techniques in the Methods section.*
☒	☐	A description of all covariates tested
☒	☐	A description of any assumptions or corrections, such as tests of normality and adjustment for multiple comparisons
☐	☒	A full description of the statistical parameters including central tendency (e.g. means) or other basic estimates (e.g. regression coefficient) AND variation (e.g. standard deviation) or associated estimates of uncertainty (e.g. confidence intervals)
☐	☒	For null hypothesis testing, the test statistic (e.g. *F*, *t*, *r*) with confidence intervals, effect sizes, degrees of freedom and *P* value noted<br>*Give P values as exact values whenever suitable.*
☒	☐	For Bayesian analysis, information on the choice of priors and Markov chain Monte Carlo settings
☒	☐	For hierarchical and complex designs, identification of the appropriate level for tests and full reporting of outcomes
☒	☐	Estimates of effect sizes (e.g. Cohen's *d*, Pearson's *r*), indicating how they were calculated

Our web collection on statistics for biologists contains articles on many of the points above.

Software and code

Policy information about availability of computer code

Data collection	All analyses were performed using previously published or developed tools, as indicated in the methods section. No custom software was used to collect the data in this study.
Data analysis	- single-cell RNA-sequencing: Computational analysis of the sequenced samples was done with Cell Ranger and the Seurat package v.3 (10x Genomics lnc.). Mapping and preprocessing were done with Cell Ranger default parameters version 3.0.2. We estimated the phase of each cell by assigning a score based on the cell expression of G2/M and S phase markers using the “CellCycleScoring” Seurat function. UMI counts were normalized using scTransform. We built a common latent cell representation across samples by integrating the sample-wise top 50 cell principal components based on the tap 1000 highly variable genes using the Seurat CCA method.). We clustered cells by first constructing a Shared Nearest Neighbor (SNN) Graph based on the Euclidean distance in the first 20 integrated principal components space using the “FindNeighbors” function with k.param set to 20. Cell clusters were defined using the Louvain algorithm as a modularity optimization technique implemented in the function “FindCluster” with the resolution parameter set to 0.2. Visualization of gene expression was computed after a new scTransform normalization run on the merged raw count assays regressing out for cell cycle and sex effect as previously described. For each cluster, conserved markers between mutant and wild types were identified using the Seurat “FindConservedMarkers” function and were then used for cell-type annotation.<br><br>- Bulk RNA-sequencing: Reads were mapped to the mouse reference genome (mm10) using the STAR mapper(splice junctions based on RefSeq; options: –alignIntronMin 20–alignIntronMax 500000–outFilterMultimapNmax 5–outFilterMismatchNmax 10–outFilterMismatchNoverLmax 0.1). Reads were subsequently used for expression analysis via the Cufflinks package (version 2.2.1; default settings). Transcripts of each sample were assembled using Cufflinks provided with reference gene annotations from Ensembl. The resulting assemblies were then merged via Cuffmerge. Heatmap results were visualized with the R package pheatmap.

- ChIP-sequencing: Reads were mapped to the mouse reference genome (mm10) using bowtie2 mapper. SAMtools was used for filtering, sorting, and removing duplicates, and deepTools for generating coverage tracks.

- 4C-sequencing: Reads were pre-processed and mapped to the mm10 reference genome using BWA. The viewpoint and adjacent fragments 1.5 kb upstream and downstream were removed and a window of 10 fragments was chosen to normalize the data per million mapped reads (RPM).

- Capture-HiC: Read mapping and further filtering for each library were carried out separately using the HiCUP pipeline v0.8.3 with Bowtie2 v2.5.0 as the aligner and no size selection or filling. Binned and Knight-Ruiz (KR) normalized contact matrices from merged biological replicates were then generated with Juicer tools v1.22.0 for the region of interest. Subtraction and matrices visualization at 5kb resolution were done using a custom Python script via the FANC Python API (available on GitHub (https://github.com/mikstapes/JGlaser-etal_Dac1J/).

- DNA methylation bisulfite-cloning- sequencing data were analyzed with the biQ Analyzer software (version 2.02, 2008)

-CTCF motif analysis was performed using the FIMO (Find Individual Motif Occurrences), MEME suite 5.5.4 (https://meme-suite.org/meme/tools/fimo)

- Image analysis was done using Fiji (Image J, version 2.1.0/1.54f, 2020)

For manuscripts utilizing custom algorithms or software that are central to the research but not yet described in published literature, software must be made available to editors and reviewers. We strongly encourage code deposition in a community repository (e.g. GitHub). See the Nature Portfolio guidelines for submitting code & software for further information.

Data

Policy information about availability of data

All manuscripts must include a data availability statement. This statement should provide the following information, where applicable:

- Accession codes, unique identifiers, or web links for publicly available datasets
- A description of any restrictions on data availability
- For clinical datasets or third party data, please ensure that the statement adheres to our policy

All datasets generated in this study have been deposited in the Gene Expression Omnibus (GEO) database and are accessible under accession code GSE246755 (containing SubSeries GSE246750, GSE246751, GSE246752, GSE246753, GSE246754). Previously published data used in this study are accessible under GSE185774, GSE116794 and GSE84795. Data were mapped to the Mus musculus mm10 genome.

Research involving human participants, their data, or biological material

Policy information about studies with human participants or human data. See also policy information about sex, gender (identity/presentation), and sexual orientation and race, ethnicity and racism.

Reporting on sex and gender	N/A
Reporting on race, ethnicity, or other socially relevant groupings	N/A
Population characteristics	N/A
Recruitment	N/A
Ethics oversight	N/A

Note that full information on the approval of the study protocol must also be provided in the manuscript.

Field-specific reporting

Please select the one below that is the best fit for your research. If you are not sure, read the appropriate sections before making your selection.

☒ Life sciences ☐ Behavioural & social sciences ☐ Ecological, evolutionary & environmental sciences

For a reference copy of the document with all sections, see nature.com/documents/nr-reporting-summary-flat.pdf

Life sciences study design

All studies must disclose on these points even when the disclosure is negative.

Sample size	Samples size was determined according to previous knowledge from the lab and the community. For expression analysis (RNA and protein), a minimum of 2 biological replicates were used per genotype and per developmental stage to unsure reproducibility. For phenotype, a larger number of samples were used to allow for detecting non-fully penetrant phenotype which would occur in 10% of the mice.<br>IF, TEM, WISH, and LacZ staining experiments were performed from at least 3 independent biological mouse embryos. FISH experiments were performed from 2 independent biological mouse embryos. Skeletal preparations were performed using at least 3 independent biological

mouse embryos or pups. Micro-CT was performed from 2 adult mice per genotype. Phenotypic evaluation of the limbs were performed using at least 60 limbs (15 animals) per experiment. Capture Hi-C experiments were performed using 2 or 3 biological replicates or 2 biological and 2 technical replicates per experiment. RNA-seq analyses were performed using 3 biological replicates. scRNA-seq experiments were performed from one biological replicate.

Data exclusions

Samples/animals were included/excluded according to the genotype.

Replication

All experiments, except for scRNA-seq which used only 1 replicate per genotype, were replicated at least 2 times.

Randomization

When generating a mouse line, two founder animals for each mouse line were used for establishing line stock with variable intercrosses between single founder and 129sv wild-type animals. If more founders were generated, those two founder were randomly selected as long as they could transmit the mutation to the F1 (germline transmission).
For allocation of samples into experimental groups, this was done according to their genotype (WT or mutants).

Blinding

Investigators were not blinded during experiments, the data collection was performed according to the stage of each sample since mouse breeding and analysis required knowledge about the genotype at hand.

Reporting for specific materials, systems and methods

We require information from authors about some types of materials, experimental systems and methods used in many studies. Here, indicate whether each material, system or method listed is relevant to your study. If you are not sure if a list item applies to your research, read the appropriate section before selecting a response.

Materials & experimental systems

n/a	Involved in the study
☐	☒ Antibodies
☐	☒ Eukaryotic cell lines
☒	☐ Palaeontology and archaeology
☐	☒ Animals and other organisms
☒	☐ Clinical data
☒	☐ Dual use research of concern
☒	☐ Plants

Methods

n/a	Involved in the study
☐	☒ ChIP-seq
☒	☐ Flow cytometry
☒	☐ MRI-based neuroimaging

Antibodies

Antibodies used

primary antibody anti-MusD-Gag was a gift from the Heidmann lab (Ribet et al. 2007) (25058 J77)
primary antibody anti-Cleaved-Caspase 3 (rabbit polyclonal, Cell Signaling Technology Cat#9661, Asp175)
primary antibody anti-gamma-H2AX (rabbit monoclonal, Cell signaling Technology Cat#9718, Ser139 20E3)
secondary antibody anti-rabbit Alexa-fluorophore 488 (Invitrogen #A11008)
secondary anntibody anti-rabbit Alexa-fluorophore 568 (Invitrogen #A110042)

Validation

The anti-MusD-Gag was previously tested by the Heidemann lab (Ribet et al 2007). When we received it, we tested its reactivity on HeLa (human), HEK-293 (human) and Neuro2A (mouse) cells transfected with a MusD expressing plasmid (phCMV-RU5-musD-6 from Ribet et al. 2004, gift from the Heidemann lab). We tested the anti-MusD-Gag antiibody (25058 J77) alongside with a negative control (serum from the same rabbit, 25058 J0) which did not show any signal.
The anti-Cleaved-Caspase3 and anti-gamma-H2AX antibodies were purchased from Cell signaling where the antibodies were certified as validated for immuno-fluorescence application in mouse samples and were cited by respectively 99 and 16 scientific papers for this application. The antibodies were first tested in wild-type samples were no apoptosis and DNA damage are expected to validate the lack of background staining.

Eukaryotic cell lines

Policy information about cell lines and Sex and Gender in Research

Cell line source(s)

We used mouse embryonic stem cells (mESCs) from 129/SvxC57BL/6J F1 hybrid (G4) background. These cells were obtained from Dr. Anders Nagy (George et al., 2007). mESCs from the Dac1J-129sv background were derived in house from bastocyst. CD1 and DR4 feeder cell lines, produced from CD1 and DR4 transgenic embryos, were used to culture the G4 cells.

Authentication

Genetically modified mESCs were used to produce embryos using tetraploid and diploid aggregation. Genotyping confirmed the presence of the desired mutation. The pluripotent state of the ESCs used was authenticated by generation of highly chimeric, germ-line transmitting mice.
CD1 and DR4 mouse embryonic fibroblast (feeder) cell lines were not authenticated. They were directly produced from mouse embryos originating from DR4 and CD1 mice crosses, respectively.

Mycoplasma contamination

All the cell lines were tested and were negative for mycoplasma contamination.

Commonly misidentified lines
(See ICLAC register)

No commonly misidentified cell lines were used.

Animals and other research organisms

Policy information about studies involving animals; ARRIVE guidelines recommended for reporting animal research, and Sex and Gender in Research

Laboratory animals	Mice from CD1, C57BL/6J, 129s2/Sv, or 129s2/SvxC57BL/6J hybrid backgrounds were used in our study. Males and females from embryonic days E9.5-E12.5 and E18.5 were used in our experiments. Routine bedding, food, and water changes were performed. Mice were housed in a centrally controlled environment with a 12-h light/12-h dark cycle, temperature of 20-22.2 Celsius , and humidity of 30-50%. All animal experiments followed all relevant guidelines and regulations.
Wild animals	This study did not involved wild animals.
Reporting on sex	Sex was not part of the study design.
Field-collected samples	This study did not involve samples collected from the field.
Ethics oversight	All animal procedures were conducted as approved by the local authorities (LAGeSo Berlin) under license numbers G0243/18, G0176/19, and G0098/23.

Note that full information on the approval of the study protocol must also be provided in the manuscript.

Plants

Seed stocks	*Report on the source of all seed stocks or other plant material used. If applicable, state the seed stock centre and catalogue number. If plant specimens were collected from the field, describe the collection location, date and sampling procedures.*
Novel plant genotypes	*Describe the methods by which all novel plant genotypes were produced. This includes those generated by transgenic approaches, gene editing, chemical/radiation-based mutagenesis and hybridization. For transgenic lines, describe the transformation method, the number of independent lines analyzed and the generation upon which experiments were performed. For gene-edited lines, describe the editor used, the endogenous sequence targeted for editing, the targeting guide RNA sequence (if applicable) and how the editor was applied.*
Authentication	*Describe any authentication procedures for each seed stock used or novel genotype generated. Describe any experiments used to assess the effect of a mutation and, where applicable, how potential secondary effects (e.g. second site T-DNA insertions, mosiacism, off-target gene editing) were examined.*

ChIP-seq

Data deposition

☒ Confirm that both raw and final processed data have been deposited in a public database such as GEO.

☐ Confirm that you have deposited or provided access to graph files (e.g. BED files) for the called peaks.

Data access links<br>*May remain private before publication.*	https://www.ncbi.nlm.nih.gov/geo/query/acc.cgi?acc=GSE246751 (reviewers token: qrofmegqzlmljgl)
Files in database submission	GSM7876362 ChIPseq_CTCF_WT_E10.5_FLHL_Rep1<br>GSM7876363 ChIPseq_CTCF_WT_E10.5_FLHL_Rep2<br>GSM7876364 ChIPseq_CTCF_Dac1J129sv_E10.5_FLHL<br>GSM7876365 ChIPseq_CTCF_Dac1JBl6_E10.5_FLHL<br>GSM8641800 ChIPseq_CTCF_WT_E11.5_FLHL_Rep1<br>GSM8641801 ChIPseq_CTCF_WT_E11.5_FLHL_Rep2<br>GSM8641802 ChIPseq_CTCF_Dac1J129sv_E11.5_FLHL_Rep1<br>GSM8641803 ChIPseq_CTCF_Dac1J129sv_E11.5_FLHL_Rep2<br>GSM8641804 ChIPseq_CTCF_Dac1JBl6_E11.5_FLHL_Rep1<br>GSM8641805 ChIPseq_CTCF_Dac1JBl6_E11.5_FLHL_Rep2<br>GSM8641806 ChIPseq_CTCF_5LTR-LacZ_E11.5_FLHL_Rep1<br>GSM8641807 ChIPseq_CTCF_5LTR-LacZ_E11.5_FLHL_Rep2
Genome browser session<br>(e.g. UCSC)	https://genome-euro.ucsc.edu/s/Juliane%20glaser/Glaser_et_al_CTCF_ChIPseq

Methodology

Replicates	n=2 biological replicates were used for WT samples.
Sequencing depth	At least 20 millions read per samples were sequenced, using single-end sequencing.
Antibodies	CTCF antibody (C15410210; Diagenode)

Peak calling parameters	Not applicable.
Data quality	Not applicable.
Software	Reads were mapped to the mouse reference genome (mm10) using bowtie2 mapper. SAMtools was used for filtering, sorting, and removing duplicates, and deepTools for generating coverage tracks.
